# Metal complexes as therapeutic substitute in tuberculosis treatment

**DOI:** 10.1186/1471-2334-14-S3-P29

**Published:** 2014-05-27

**Authors:** C Justin Dhanaraj, M Sivasankaran Nair

**Affiliations:** 1 Department of Chemistry, University College of Engineering Nagercoil, Anna University, Tirunelveli Region, Tamil Nadu, 629004, India; 2 Department of Chemistry, Manonmaniam Sundaranar Universiy, Tirunelveli, Tamil Nadu, 627012, India

## Background

Tuberculosis (TB) is a disease that produces several million deaths annually. With the appearance of multi drug resistant microbial strains of *Mycobacterium tuberculosis*, innovations in TB drug discovery and evolving strategies to bring new agents with best performance is an essential investigation. Taking this into account, there is a pressing need to develop new and more effective anti tubercular agents. The coordination of metal with organic drugs is a promising strategy that has been successful in many cases with different pharmacological activities. The emergence of new cases and the adverse effects of first and second-line antituberculosis drugs have led to renewed research interest in metal drug complexes in the hope of discovering new antituberculosis drugs. The aim of the present study is to assess the antituberculosis activity of Ni (II) and Cu (II) complexes of polymer ligand poly (3-nitrobenzylidene-1-naphthylamine-co-methacrylic acid).

## Methods

Antituberculosis activities of the ligand and its complexes were assessed against *M. tuberculosis* H37 Rv strain by microplate alamar blue assay (MABA) method using pyrazinamide as standard.

## Results

The minimum inhibitory concentration values of the ligand and its complexes on comparison with standard (pyrazinamide; 4 µg/mL) indicate that the complexes exhibit promising antituberculosis activity. Cu (II) complex shows more activity (10 µg/ mL) followed by Ni (II) complex (20µg/ mL) and ligand (35 µg/ mL).

## Conclusion

Among the synthesized compounds, the Cu (II) complex may be used as therapeutic substitute in the treatment of tuberculosis by replacing the routine drugs.

